# An exploration of nursing and Allied Health Professional (AHP)-led post-treatment surveillance and survivorship care for people with head and neck cancer—a scoping review

**DOI:** 10.1007/s00520-025-09272-5

**Published:** 2025-03-13

**Authors:** Sinead Rothrie, Grainne Brady, Paul Howell, Justin Roe

**Affiliations:** 1 Department of Speech, Voice and Swallowing, The Royal Marsden NHS Foundation Trust, London, UK; 2 Department of Surgery & Cancer, Imperial College London, London, UK; 3 The David Adams Library, the Royal Marsden NHS Foundation Trust, London, UK

**Keywords:** Scoping reviews, Advanced practitioners, Allied health professionals, Head and Neck cancer, Cancer surveillance, Survivorship

## Abstract

**Abstract:**

A scoping literature review was undertaken to identify the current evidence base on the role of nursing and allied health professionals (AHPs) in delivering surveillance and survivorship services within head and neck cancer (HNC) care following treatment.

**Method:**

This review was undertaken according to the Joanna Briggs Institute (JBI) guidance on the conduct of scoping reviews. An initial database search was undertaken between December 2023 and February 2024 and then repeated in November 2024. Databases included CINAHL, EMBASE, and MEDLINE. A focused grey literature search targeting other material including conference abstracts was also completed. Articles were included which were written in English. The search was not restricted to year of publication/production or methodology to ensure the greatest scope of materials. Relevant articles were reviewed, and narratives summarised.

**Results:**

A total of 144 articles were identified through initial database screening and subsequently 29 were eligible for full text review with 3 meeting the inclusion criteria. All 3 investigated follow-up care led by nurses or AHPs for people treated for HNC. Two of these articles described alternative models of surveillance/survivorship care. One article aimed to investigate professional’s perceptions on post-treatment disease surveillance by nurses and AHPs. Outcome measures included quantitative results on quality of life measures (QoL) and disease re-sectability and qualitative data obtained via an online survey which included free text response options. Limited results demonstrated that people were satisfied that nurse or AHP led care could meet their needs and improve psychosocial adjustment and QoL. There was no evidence to suggest the rate of cancer recurrence detection is reduced when a nurse or AHP is the lead professional involved in follow up surveillance. No articles explored the experience of people receiving this model of care in detail.

**Conclusion:**

A small body of evidence suggests that nursing and AHP professionals can provide an effective and safe service of follow-up care in HNC management. Clinics led by advanced practitioners (AP) may provide an opportunity to deliver enhanced care and meet QoL needs. Within a rapidly developing and changing landscape of post-treatment surveillance nurses and AHPs are well placed to provide advice, support and interventions for treatment effects. More evidence is needed to develop new models of risk stratified nursing/AHP surveillance and the competencies required to ensure the complex holistic needs of individuals are safely and effectively met.

## Background

Head and neck cancers (HNC) are located in the upper aerodigestive tract. There has been a significant change in how it is diagnosed and treated over the past two decades. Incidences of the disease continue to rise worldwide [1] with traditional risk factors including smoking and alcohol-related factors continuing as persistent root causes. There is also the increase of human papilloma virus (HPV)-related disease [2]. Radiotherapy, chemotherapy and surgery are all used to treat HNC, often combined as multi-modality treatments [3]. HNC and the anti-cancer treatments provided can have a significant impact on an individual’s ability to eat, drink, swallow and communicate [4]. The acute, long-term and late impact of treatment on swallowing has been well documented in the literature [5, 6]. Consequently, and despite ongoing advances in treatment techniques, HNC causes unique QoL challenges which impact on longer term outcomes related to living with and beyond cancer.

Following treatment people are followed up for a period of monitoring and surveillance. Historically, the primary role of this follow-up care was to ensure there was no return of the original disease and this was solely the realm of oncology and surgical teams [7]. The traditional design of such follow-up is built on the premise that the likely recurrence will be logo-regional and as such close monitoring within the first few years may allow for surgical salvage if deemed necessary [8]. However, surveillance which aims to recognise only the signs and symptoms of recurrence, may overlook the holistic QOL concerns of some [9].

Across other tumour sites there is an increasing understanding that people may benefit from a more personalised, holistic approach to follow up care [10] focusing not only on the presence or absence of disease, but on the survivorship needs of the individual which nurses and allied health professionals (AHPs) may be best placed to offer. More people are living beyond treatment and there is an onus on health professionals to ensure that the QoL experienced is considered, evaluated, and supported in the context of their post-cancer needs [11]. The lens on solely oncological-focused outcome measures is no longer meeting the complex and varying QoL challenges that many people experience [12].

The concept of survivorship was first described in 3 phases (acute, extended and permanent) in 1985 [13] although definitions and use continue to be inconsistent [14, 15]. Within the HNC cohort it is well-documented that the known sequelae of treatment can have ongoing impacts on patient’s QoL symptoms including, but not limited to, reduced mobility, eating and drinking difficulties, psychosocial concerns and intimacy [16]. It is in this context, of supporting and optimising functional QoL needs within the post treatment phase, that the term survivorship will be used in this paper. With a greater focus on survivorship there is increased acknowledgement that healthcare professionals should be doing more to support people after treatment and helping to shape what life beyond treatment may look like [9, 17–19]. Additionally, within many tumour groups and specifically within HNC, there is increased knowledge around the late effects of treatment [20]. The definition of late effects is not conclusive however it has been defined as ‘unrecognised toxicities that are absent or subclinical at the end of therapy’ [21] and can be widely variable dependant on the tumour type and staging, mode of treatment as well as the patient’s health status [22]. Our current understanding of late effects in HNC is that of persistent negative side-effects from treatment which, following a period of stability, have increased and are having a significant impact on day-to-day functioning [18]. People can experience late toxicities from months to many years following their treatment [23, 24].

Patterns of follow-up are changing in response to the growing number of HNC survivors. There is increasing recognition that traditional models are unsustainable and may not address unmet needs [25]. In addition, the rising number of people being diagnosed with new HNC means the current model of cancer detection and surveillance may not be sustainable. As most individuals with recurrence present with a change in symptoms, ongoing medical examination may also be unnecessary [26]. Current research within the UK is examining the use of PET-CT guided, person-led follow-up compared to the current standard of care (SOC) [27]. This research is investigating the feasibility of low-risk individuals stratified into a pathway, which does not routinely offer follow up appointments. Studies have also examined the role of biomarkers to risk-stratify routine follow-up [28]. Both areas of work are aiming to increase the efficiency of follow up to reduce unnecessary appointments for those at low risk of cancer recurrence. However, it is important not only to think about efficiency of follow up, but the value and importance of individuals QoL and function. Those people who are at lower risk of cancer recurrence, may not be at lower risk for treatment effects.

In the United Kingdom's National Health Service (NHS), there is a drive for models of care to innovate and utilise the skills of other health professionals, particularly Advanced Practitioners (AP). Advanced practice allows AHPs to work in expanded roles to better serve the populations and clinical settings in which they work. This will often take the form of extended scope of practise, utilising skills and experience to service a clinical caseload [29]. The goal is to ensure the delivery of quality care as well as to release and support capacity in the system. Within the NHS Long-Term plan and new Aspirant Cancer Career and Education Development programme (ACCEND) programme (which seeks to provide a professional framework for nursing and AHPs within cancer care) there are strategies for the expansion and utilisation of advanced and specialist staff [30, 31]. These initiatives benefit both staff and those receiving care with improved waiting times and satisfaction as well as increased staff engagement and retention. There is increasing understanding of the key roles that nursing and AHP professionals can have in the re-design of services to meet individuals’ clinical and psychosocial needs [32]. Implementation of AP roles within nursing and more recently, AHPs, have been in development for the past few decades [33] and need to expand further [32]. Within cancer surveillance, there is a body of evidence which support AHP and nursing led care [16, 34–36] across a range of tumour groups, however this has not been widely explored within HNC.

The extended scope/AP model of nursing and AHP-led cancer care is critical to post treatment survivorship and satisfaction, and it has been previously demonstrated that effective and timely provision of support can be of benefit to individual’s outcomes [37]. It is therefore essential to understand how these skills are currently being utilised to support current surveillance and survivorship models of care within HNC.

## Aim

The aim of this review was to examine the evidence base for the role of nursing and AHPs in leading surveillance and survivorship clinics within HNC care, to establish current models of care, and the measures currently in use to demonstrate outcomes in terms of efficacy and experience. Literature was reviewed on the creation of these clinics where medical professionals were not the lead clinician.

## Methods

The research question for this review, was developed by two authors SR and GB with the aim of identifying the current literature pertaining to the lead role of nursing and AHPs in leading survivorship and surveillance within HNC care.

### Design

**Fig. 1:**
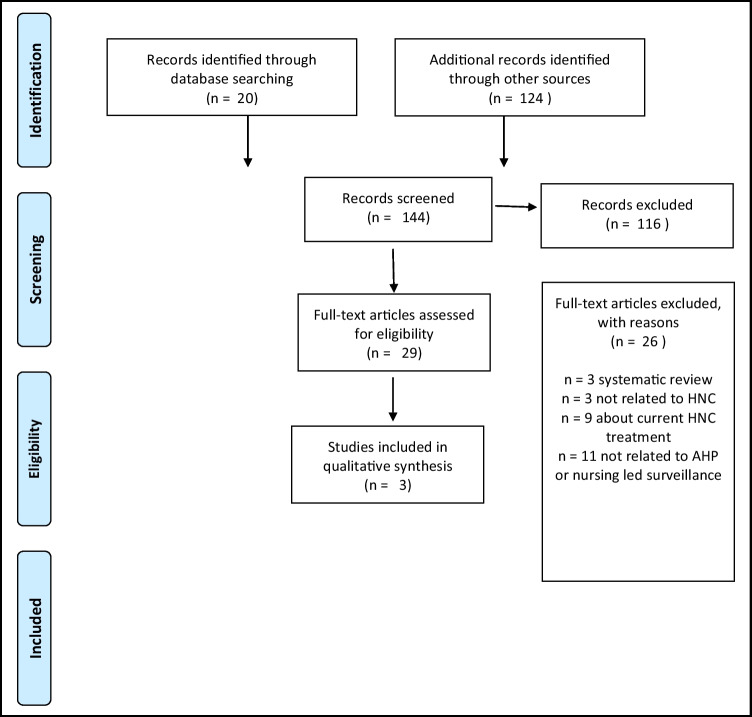
PRISMA flow diagram [41]

This review was designed using a strategy developed originally from Arskey and O'Malley [38] and latterly added to from Levac et al. [39]. It was informed by the Joanna Briggs Institute (JBI) [40] and includes the use of the PRISMA checklist [41] (Fig. 1).

### Search strategy

An academic librarian (PH) was consulted throughout the process, in particular to support the development of a search strategy and acquisition of papers. An initial search of CINAHL returned results pertaining to the topic and this was used to refine and agree upon the full keyword search terms and MeSH / subject terms to be used where appropriate. These included: “Head and Neck neoplasms” AND “Late effects”, AND “AHP”, AND Radiotherapy, AND Survivorship. The search took place between December 2023 to February 2024 and was repeated in November 2024 to ensure any newer materials were included.

All search terms were exploded when possible and any subject headings relevant to each database were included. The keywords were checked with author GB and any disagreements resolved through discussion. Support was also sought from PH to check the inclusion or exclusion of criteria was appropriate as well as the introduction of grey literature.

Using the identified search strategy, searches were conducted in CINAHL, EMBASE, MEDLINE using appropriate subject headings / MeSH headings where variations occurred between databases. The results were downloaded as csv files and stored in a Microsoft Excel Spreadsheet. The spreadsheet was subsequently ordered alphabetically, and duplicates deleted [42]. The full text articles were obtained, and the reference lists of articles were also reviewed to ensure there was no additional papers that had been missed in the full search as well as a full citation search of selected articles.

Additionally, a grey literature search was conducted to ensure that materials related to this knowledge area were included. An initial Google search was conducted replicating the keywords in the database searches as the authors were aware of work being presented at national conferences in related fields. All returned searches (pages 1–4) of Google results were checked until no new sources were identified. Although never to be used as a standalone source of searching, grey literature is an important addition to search strategies [43] as materials within other sources can provide information on topics where there is paucity of good quality evidence and can help to share future research priorities [44].

### Inclusion terms

There was no time range included, and papers were sought which explored the role of nursing and/or AHP professionals in the surveillance or provision of survivorship care for people who are finished treatment for HNC. Following initial searches and the lack of available evidence collated papers were included which examined models of care as well as those seeking to understand perceptions of nursing and AHPs role in surveillance care. Inclusion criteria is detailed in Table 1.

**Table 1:** Inclusion criteria

R1	Is related to head and neck cancer squamous cell carcinoma (SCC)
R2	Involves adults (> 18 years)
R3	Does not include current toxicities during active cancer treatment
R4	Is written in English
R5	Does not include individuals receiving active or trial treatments
R6	Is related to nursing or AHP led follow up

### Exclusion criteria

Papers were excluded which did not include full text articles.

### Data extraction and analysis

A data extraction table was devised by the lead author within Microsoft Excel [42]. Data inputted included journal title, authors, location, purpose, methods, outcome measures if appropriate and main findings. Themes and topics which emerged form the basis for this review. These themes were discussed with another author (GB) and agreements made that the summaries and representation of content was accurate. Any discrepancies between authors views were discussed in person to ensure agreement was reached. Given the limited number of studies included in the final results there was no disagreement about selection.

## Results

In total, 20 articles were identified in keyword searches and 124 from other sources including reference and citation list searching and grey literature searches. Abstracts were screened in the total 144 articles. The abstracts were assessed for eligibility against the screening criteria outlined previously. This resulted in the identification of 29 articles for full text review. Following full text review against the inclusion criteria, three articles were included in the review (Fig. 1).

De Leeuw et al. [33] reports on a nurse-led follow-up care for people treated for HNC comparing conventional SOC to follow-up with an addition focus on survivorship factors. Silva Nash et al. [45] report retrospective analysis of recurrence rates at an Advanced Practice Provider-Led (APPL) HNC follow-up clinic in the USA and Wells et al. [46] discusses a study which explored nurse and AHP views and practice in relation to follow up and survivorship care in HNC through the use of a questionnaire sent out to British Association of Head and Neck Nurses (BAHNON) members (Table 2). In order to identify common themes, thematic review analysis was conducted whereby articles were thoroughly read to ensure the author was familiar with the materials, before being reviewed to identify key themes which accurately capture the essential findings. Findings have been grouped under four main themes, monitoring and detection of recurrence, psycho-social factors, nursing and AHP confidence/competence, clinical outpatient examinations.

**Table 2:** Qualitative and quantitative research findings

Author	Location	Date of Publication	Participants	Aims	Study design	Methods	Outcome measures used and timepoints	Main Findings
De Leeuw et al. [33]	UK	2013	160	Compare conventional medical follow up with follow up with additional nursing consultation	Single centre Patients enrolled in consecutively in two groups (comparison and intervention group)	Standard of care vs intervention group of nurse-led follow up Seen 6 follow up appointment (over 12 months)	EORTC QLQ C30 PAIS-SR 1,6, 12 months	Improved quality of life symptom scores (and an increase in psychosocial adjustment scores however not statistically significant
Silva Nash et al. [45]	USA	2022	570	Report on the efficacy and safety of an Advanced Practice Provider-led (APPL) HNC survivorship clinic	Single centre Retrospective review of patient electronic records	Retrospective quantitative analysis from patient records Clinic consistent with established guidelines [47] Computer tomography imaging and laboratory work alongside a oral history and physical examination. Flexible laryngoscopy and biopsies were taken as indicated	Number of patients diagnosed with surgically re-sectable disease recurrence over 46 months Margin status of salvage surgical resection for local recurrence and for regional recurrences; extra-nodal extension Reason for lost to follow up status	88.9% of recurrences being locoregional and qualifying for curative intent salvage surgical interventions The average time to recurrence from initial completion of treatment was 39.1 months 12 new secondary cancers were identified
Wells et al. [46]	UK	2015	74 (45% response rate)	Explore views and practices of healthcare professionals of models of follow up and survivorship care for HNC patients in UK	An online survey emailed to British Association of Head and Neck Cancer Nurses (BAHNON) database anonymously	Included sections on; current practise and view of follow up care, practise, views and experiences of Holistic Needs Assessments (HNA), practise in care planning, provision of rehabilitation support and the confidence to provide post treatment management support	Number of respondents	65% (n = 48) were clinical nurse specialist (CNS) and most worked in cancer centres (39%) 70% of respondents felt either nurses or AHPs could conduct follow up for the patients (70%) Free text responses were included which need for adequate training skills to perform roles

### Monitoring and detection

Within Silva Nash et al. [45], clinic visits were scheduled consistent with established guidelines [47] and demonstrated that detection rates (10.7%) correlated with other research [48] with 88.9% of recurrences being locoregional and qualifying for curative intent salvage surgical interventions which did or did not include adjuvant therapies. The average time to recurrence from initial completion of treatment was 39.1 months (range 12–176 months). In the cases of 12 people, a secondary primary cancer was detected indicating the screening tools were appropriate. It was suggested within Wells et al. [46] that there is a tendency within cancer risk-stratification for the focus to be solely on the risk of recurrence and that it should also consider the identification of after-effects of treatment at earlier points. This would allow the possible recognition of those most at risk of development longer term complications.

### Psycho-social factors

De Leeuw [33] used primary outcome measures were psychosocial adjustment (Psychosocial Adjustment to Illness Scale – Self reported PAIS-SR) and QoL (The EORTC QLG Core Questionnaire (EORTC QLQ-C30) with people completing outcomes scoring at 1 month, 6 month and 12 months post treatment. These outcome measures were not taken prior to treatment. The overall response rate was 78%. The mean scores at 1 month for the PAIS-SR, which the authors used as the baseline, were worse in the treatment group; however at 6 months, there was no significant difference between the two groups. Likewise, HRQoL were significantly worse at baseline in the intervention group and similar to the comparison group at 6 and 12 months, indicating a larger overall improvement in scoring between the two groups. The largest improvements were seen for fatigue (6 months) and pain and social eating (12 months). The improvements were not statistically significant. When indicated, the use of an assessment tool for depression and anxiety was administered in the Silva Nash study however the results were not shared [45] and so no conclusions from these results can be drawn. Through the methodologically different collection of results via an online questionnaire Wells et al. [46] gathered responses from nurses and AHPs on their use of psycho-social assessment tools. This demonstrated 1/3 of those who responded used the Health Needs Assessment (HNA) and 36% used it during post treatment care; however, respondents were just 43% of total membership and heavily weighted towards nursing. When they were asked their preferences of which tool they would like to use 60% chose the Patients Concerns Inventory (PCI), perhaps suggesting the current established tools need more investigation.

### Nursing and AHP confidence/competence

De Leeuw [33] stated that prior to recruitment nurses participated in specific training in biopsycho-social models of care and communication skills as well as a session from HNC surgeons on completing simple medical checks. It was reported most consultations could be performed adequately within 30 min and nurses indicated increased work satisfaction highlighted with verbal responses in the paper. The topic of confidence in skills or experience was not explored within Silva Nash’s paper, as a retrospective review it focuses on the analysis of recurrence rates rather than qualitative findings; however, it was noted that there could be barriers to other institutions adopting this clinic design if specialised training for staff on detecting recurrence was not available. Within the questionnaire [46], the majority of those who responded (70%) concluded nurses and AHPs could conduct follow-up for HNC patients. Conversely, it was also acknowledged through free text responses that training with advanced skills and onsite support from medical teams would be necessary. The findings demonstrated many respondents would be keen on more responsibilities but there are specific areas of practise that nurses lack confidence, knowledge and skills. Most felt confident in providing support on the commonly reported symptoms such as dry mouth, pain, taste, fatigue, chewing, and eating as well as smoking cessation; however, 1/3 were less confident in offering support with sexual difficulties, lymphoedema, dental, work and financial related issues.

### Clinical outpatient examinations

Nurses saw patients prior to their medical appointments and conducted simple medical checks within the first paper [33] including palpation of the neck and oral cavity and inspection of tracheal stoma, cannula, and speech valve where appropriate. In 49% of consultations, the nurses completed the medical checks independently and in 37% these were performed by the clinician often due to the fact the patient required laryngoscopy and therefore to minimise burden on the patient all the checks were completed by the physician. In the APPL led clinic [45] imaging and lab work was taken at every visit alongside interval history, a physical examination including flexible laryngoscopy and biopsies as indicated. Those who had received adjuvant therapy continued to be seen in a separate appointment by oncologist.

## Discussion

The aim of this scoping review was to examine the current evidence pertaining to nursing and AHP-led follow-up care in HNC. Three articles were included. The article by de Leeuw and colleagues [33] specifically investigated the impact of additional survivorship support provided by nursing staff to people alongside their standard of care medical led surveillance clinic using a quasi-experimental approach. Although the groups were not matched in terms of psychological adjustment and HRQoL baseline scores, the scores appeared to become comparable at 6 months. The authors concluded that given the lower scores recorded in the treatment group at baseline, nursing input focusing on survivorship follow-up can enhance psychological adjustment and improve overall QoL.

The article by Silva Nash and colleagues [45], focused on cancer surveillance predominantly where AP nurses led the surveillance clinic and completed the assessment in place of the standard of care surgical team follow up. Via retrospective review of the detection rate of early recurrences, identified using the surrogate of surgical resectability, the authors concluded that this is a safe model of HNC surveillance. The survivorship issues/outcomes were not reported.

The final article included by Wells and colleagues [46] investigated nursing and AHP views on follow-up pathways and their role in survivorship post HNC. Although predominately including nursing views only, respondents were open to new models of care, and greater responsibility if adequate training is provided. The survey was designed to be short and easy to access which does mean some of the information provided is open to interpretation and therefore had some limitations to its value.

It is evident from research ongoing in HNC that the current model of follow-up will be changing in the future nationally and internationally [27, 28], with increased emphasis on patient-initiated follow-up shaped through individualised choice, further education on symptoms and addressing ongoing support needs [27, 49]. From the limited findings of this review, it is proposed that AHP and nursing can have a positive impact on follow up for people treated for HNC in the provision of survivorship care.

Speech and language therapists (SLTs) working to full scope [50] as APs are already utilising their skills within endoscopic evaluation as part of the diagnostic pathway [51] and there is evidence demonstrating first contact SLT led clinics can reduce waiting lists for medical colleagues [52]; however, less focus has been given currently to how these roles can be further developed in post treatment care. Within the USA, nurse led surveillance and survivorship clinics which utilise AP skills including independent prescribing, the ordering of scans and phlebotomy results are more commonplace than within the UK [45, 53]. This is in part due to guidance which seeks to shift the attention to people’s longer-term needs and QoL, rather than surveillance for recurrence alone [19]. Whilst there is support among professional forums for the development of nursing and AHPs roles some have expressed concerns within current UK practise about developing the level of standardised competencies to provide the required range of medical tests and procedures [46]. There is acknowledgement that for the development of AHP led clinics the support of medical colleagues is imperative to their implementation [54]. There has been an increased focus on the development of services which seek to understand the management of late effects following treatment [55]. However, this does not seek to establish longer-term ongoing surveillance to monitor after-effects and develop preventative interventions to target the known sequalae’s of treatment prior to them becoming debilitating for individuals.

Within the context of nursing and AHP-led survivorship and surveillance this review demonstrates that the use of nursing and AHPs as the lead professional in survivorship clinics which focus on medical monitoring and QoL symptom management may provide increased satisfaction, have a positive impact on HRQoL scores [33] and does not result in a reduction in the detection of cancer recurrence rates [45]. Nonetheless, it must be acknowledged that the evidence base examined is limited and further work is needed to truly understand the potential impacts.

There is an important link between post-treatment follow-up, cancer monitoring, and the ongoing impact and effects of treatment on people’s QoL. If surveillance moves away from medically led follow-up there is a potential detrimental effect on the individual’s quality of overall care. Further work is required to understand and define what we mean by both survivorship and late effects in the context of surveillance clinics to better meet the needs of people diagnosed by HNC. The current understanding of survivorship does not adequately define the challenges of life beyond cancer to include the complex day to day intricacies of physical, psychological and social elements which go on to affect many people and the impact of the late effects of many treatments, including HNC. Similarly, the current understanding of the late effects of treatment in HNC is still emerging and there is not enough evidence currently which includes the experience of individuals to truly demonstrate the challenges faced as they recover from treatment and move on to the next stage of their life.

HNC survivors are a diverse and heterogenous group with a wide range of presenting cancer types and aetiologies [56], as well as differing socio-economic and socio-demographic statuses [57]. These factors will have an impact on the individual’s ability to engage in decision making and autonomy within the context of survivorship care [19]. Between the continuum of patient-initiated follow-up and profession-specific monitoring is the case for a shared care mode (which seeks to integrate either primary care and specialist provision or healthcare provider and patient/carer in shared responsibility for follow-up care). Whilst models exist across other tumour sites [58], there is little evidence of its outcomes in HNC and warrants further investigation and understanding. Most importantly, increasing our knowledge about how to include those affected as co-participants in developing the evidence base will start to build more effective models of care and interventions to meet their needs. The role for self-efficacy has been explored in other tumour groups [59] and attempts made to apply this in the HNC population through a prospective randomised controlled trial (RCT) however these results demonstrated a single intervention was not sufficient to improve QOL concerns and were not statistically significant. [60].

Involving people who are experts by experience are key when making decisions around new models of service delivery [46]. The absence of evidence of the lived experience of HNC survivorship documented in the included papers should galvanise efforts to encourage both individuals and their caregivers to contribute, be listened to and share their experience to shape what innovative service design can look like.

## Limitations

The articles discussed focus on single centre studies with high levels of bias embedded into the research. Searches were conducted in English only and difficulties with terminology and the definition of AHPs is likely to have hampered the overall quality of research found. The articles included did not seek to include the view and experience of either individuals or professionals to any great detail in contextualising the models and ways of working and were themselves methodologically different, two examining models of care and one seeking opinions and perceptions of working models. One of the studies was based in the USA with a different healthcare model to that of the UK which may affect how some of the practice was carried out. Given the specific search terms articles were not included where clinics recruited patients across multiple tumour sites, and this will inherently result in materials being excluded. A recent scoping review identifying models of care supporting people in rural areas with HNC adds an additional dimension to our understanding; however, this reviewed articles across the entire pathway rather than specifically within post treatment settings [61]. Grey literature searches whilst relevant are inherently less rigorous and therefore results are provided with caution. The scope of this review and its search criteria also precluded the inclusion of late effects clinics which are predominately managed by radiographers [62] as these clinics tend to treat people across tumour groups. However, there is relevance when discussing the extended practice of AHPs.

## Conclusion

Longer term monitoring and surveillance for people after treatment for HNC is routinely conducted by medical professionals within unidisciplinary clinics. Although there is an understanding that medical capacity is under pressure and new ways of monitoring people for cancer recurrence is ongoing, there is not yet evidence available which demonstrates robust innovative new models of care which seek to change this model of service delivery. The move away from the current organisation may be an opportunity to offer a dual service, the surveillance of recurrent disease alongside the ongoing assessment of patients QOL and survivorship needs [8]. The instances of non-medical follow-up which have been published demonstrate clinics have the potential to provide the right information, discuss some of the ongoing challenges living beyond cancer treatment, and adequately detect recurrence. However, further prospective studies are required to properly identify the needs of the people involved and explore suitable models of care and intervention which reduce the load on routine surveillance and meet QoL needs.

## Future implications

More robust research is needed which seeks to understand, in detail, how advanced AHP practitioners can contribute to this field and provide both patient and medical colleagues with the right support and tools to empower survivorship skills in people and the right level of care for ongoing surveillance.

## Data Availability

No datasets were generated or analysed during the current study.
